# Mechanisms of Strength Degradation of Dental Zirconia Due to Glazing: Dependence on Glaze Thickness

**DOI:** 10.3390/ma18030684

**Published:** 2025-02-04

**Authors:** Kazumichi Nonaka, Mitsuji Teramae, Giuseppe Pezzotti

**Affiliations:** 1 Department of Research and Development, SHOFU Inc., Higashiyama-ku, Kyoto 605-0983, Japan; 2 Biomedical Engineering Center, Kansai Medical University, Shin-machi, Hirakata 573-1191, Japan; pezzotti@hirakata.kmu.ac.jp

**Keywords:** dental material, zirconia, glaze

## Abstract

Glazing is a common method for smoothing the surface of zirconia and imitating the appearance of natural teeth. Several authors have previously reported that glazing reduces the strength of zirconia. However, the dependence of strength on glaze thickness and the mechanism of strength reduction remains unclear. Clarifying these factors is particularly important for improving the reliability of zirconia prostheses. In this study, three types of zirconia were glazed with various thicknesses, and their strength was evaluated. The results showed that the strength of the materials decreased with increasing glaze thickness. The decrease in the fracture load of the glazed test specimen stopped at a load where the stress necessary to fracture the glaze material was applied to the surface of the glaze layer. Furthermore, the strength reduction mechanism was investigated using FEM analysis, fractography, and Raman spectroscopy. The results suggested that the strength reduction due to glazing was a consequence of the crack-tip stress concentration developed upon the preliminary fracture of the glaze layer.

## 1. Introduction

Ceramic materials have long been used as dental materials due to their high aesthetics, mechanical properties, chemical stability, and biocompatibility. Zirconia, especially, has played an important role in modern dental prosthetic treatments because it merges suitable aesthetic characteristics and superior mechanical properties [1,2,3]. Furthermore, applications of zirconia extend to dental implants, artificial hip joints, and aptasensors [4,5,6].

Since human teeth have a three-dimensional structure consisting of dentin and enamel, it is difficult to reproduce the color tone using zirconia alone. Glazing (including staining) is a common method for smoothing the surface of zirconia and imitating the appearance of natural teeth [7,8,9].

The mechanical properties of glaze materials are lower than those of zirconia, so how glazing affects the mechanical properties of zirconia is of great clinical interest. It is easy to infer that veneered zirconia, which has a larger porcelain thickness than zirconia, exhibits lower strength than monolithic zirconia. Furthermore, many other studies have shown that even glazing (having low porcelain thickness) reduces the strength of zirconia. [10,11,12,13,14,15,16].

Previous studies provide some indications about the strength reduction mechanism of zirconia due to glazing. Doğru et al. [12] showed that glazed zirconia specimens had significantly lower flexural strength than zirconia polished with polishing paste despite having a lower surface roughness. Since the zirconia surface of the glazed zirconia specimen was not polished, this result suggests that the surface roughness of the zirconia, rather than the surface roughness of the glaze layer, influences the flexural strength. Lohbauer et al. [17] summarized previous research on the effects of veneering on zirconia. Hsueh et al. [18], theoretically, showed that the stress distribution during biaxial bending of veneered zirconia differs depending on whether the veneer layer is placed on the tension side or the compression side due to the difference in the elastic modulus of each layer. Furthermore, in the same way as porcelain for metals, porcelain with a coefficient of thermal expansion (CTE) about 10% lower than that of the frame material is generally used as porcelain for ceramics. Swain et al. [19] showed that when using such porcelain, compressive stress is applied to the porcelain layer, and tensile stress is applied to the frame material. From these studies, it is assumed that stress concentration due to the difference in elastic modulus between zirconia and porcelain, residual stress in zirconia, and roughness of the zirconia surface are the causes of the low flexural strength of glazed zirconia.

On the other hand, studies by Singh et al. [13] and Hatanaka et al. [15] showed that glazing significantly reduces the flexural strength of zirconia fabricated using the same polishing protocol. This result suggests that not only the surface roughness and residual stress of zirconia but also the presence of the glaze layer itself is the cause of the decrease in the flexural strength of the test specimen. Singh et al. showed that the crack propagated seamlessly from the glaze layer into the zirconia and speculated that the brittle glaze layer facilitated fracture initiation [13].

As mentioned above, many experimental and theoretical studies have been conducted regarding glazed zirconia [10,11,12,13,14,15,16]. However, there are still some points about the effect of glazing on the strength of zirconia that are not fully understood. First, the effect of glaze thickness on strength. These studies evaluated a single glaze thickness, so the dependence of strength on glaze thickness is unclear. In clinical practice, the thickness of the glaze layer varies depending on the operator, so it is especially important to clarify the relationship between glaze thickness and strength reduction. Second, the mechanism of strength reduction. As mentioned above, previous studies suggest that the presence of the glaze layer itself is the cause of the reduction in the bending strength of zirconia. However, the mechanism of strength reduction has not been clarified in detail. Finally, the effect of zirconia type. The majority of the previous studies only investigated 3 mol% yttria-stabilized zirconia (3Y zirconia) as core zirconia material. In current clinical practice, 4 mol% yttria-stabilized zirconia (4Y zirconia) and 5 mol% yttria-stabilized zirconia (5Y zirconia) are also used depending on the case [20,21]; therefore, it is necessary to investigate the effects of glazing on these types of zirconia. As a small example, Dogru et al. [12] used four types of zirconia with different yttria concentrations in their study, but the effect of zirconia polishing made it difficult to evaluate the effect of glazing.

The purpose of this research is to clarify the influence of glaze thickness on the strength of various types of zirconia. The null hypothesis of this study is that the glaze thickness has no effect on the three-point bending strength of zirconia.

## 2. Materials and Methods

### 2.1. Preparation of Test Piece

**Figure 1:**
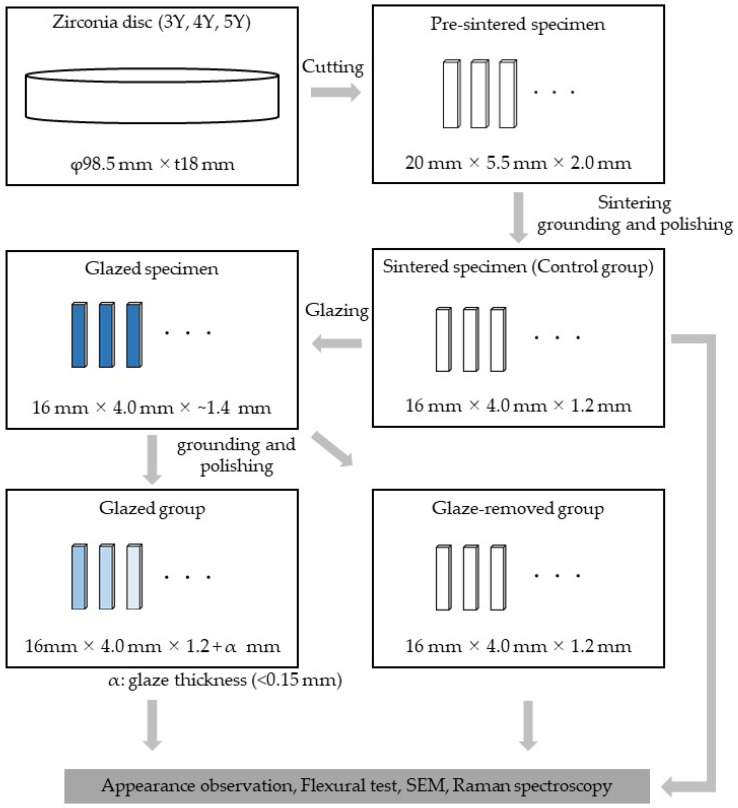
The specimen preparation procedure.

Figure 1 shows the specimen preparation procedure. Zpex, Zpex4, and Zpex smile (Tosoh, Tokyo, Japan) were used as raw material powders for the 3Y, 4Y, and 5Y zirconia sintered bodies, respectively. Various raw material powders were press-molded at 410 kN using a uniaxial press molding machine (Sansho Industry, Osaka, Japan) and CIP-treated at 200 MPa using a cold isostatic press machine (Sansho Industry, Osaka, Japan) to obtain a molded body with a diameter of 98.5 mm × 18 mm. The obtained molded body was degreased at 500 °C using an inert gas oven (INH-21CD, KOYO THERMO, Nara, Japan) and then fired at 975 °C using a firing furnace (SC-3035F, MOTOYAMA, Osaka, Japan) to obtain a zirconia-calcined body. The zirconia-calcined body was cut with a cutting machine (Secotom, Struers, Ballerup, Denmark) and polished with SiC polishing paper (#1000) to obtain a pre-sintered flexural test specimen (20 mm × 5.5 mm × 2.0 mm). The test specimen was sintered at 1500 °C (dwell time: 2 h) in a sintering furnace (AUSTROMAT 674i, DEKEMA, Freilassing, Germany) and ground using a diamond wheel in a surface grinder (GRIND—X PFG500II, OKAMOTO, Gunma, Japan) to obtain a flexural test specimen (16 mm × 4.0 mm × 1.2 mm). The surface (16 mm × 4.0 mm surface) of the test specimen was polished with SiC polishing paper (#1000) in accordance with ISO 6872 (Dentistry—Ceramic materials) [22] and then chamfered (45° edge chamfer).

Glazing was performed on the tensile surface of each flexural test specimen except for the control group. Commercially available aluminosilicate glass porcelain Vintage Art Universal GP (Young’s modulus: 61 GPa, CTE: 8.4 × 10^−6^ K^−1^, SHOFU INC., Kyoto, Japan) was used as the glaze material. Glazing material powder was diluted with a special liquid to form a paste, applied to the surface of the test specimen, and then fired according to the manufacturer’s specified firing schedule (740 °C in vacuum) to perform glazing. The glaze thickness at this point was approximately 0.2 mm. The glaze surface was ground and polished using a surface grinder and waterproof abrasive paper to prepare specimens with various glaze thicknesses. Since precise control on the micrometer order is difficult in polishing, the exact glaze thickness was obtained by observing the fracture surface after a three-point flexural test (see Section 2.2). Finally, the edges of the glazed surface were chamfered with SiC polishing paper (#1000) (45° edge chamfer). Using a similar procedure, a glaze-removed group was prepared, and the glaze layer was completely removed by polishing in order to evaluate the chemical influence of the glaze treatment. The sample size for the control group and the glaze-removed group was n = 10 according to ISO 6872 [22].

### 2.2. Three-Point Flexural Test

Three-point flexural tests were performed on the control group (without glazing: n = 10), the glaze-removed group (after glazing, the glaze layer was removed by polishing: n = 10), and the glazed group (n = 72 (3Y), 48 (4Y), and 45 (5Y), respectively). The test was conducted in accordance with ISO 6872 (Dentistry—Ceramic materials) using a universal testing machine (Instron 5967, INSTRON, Norwood, MA, USA) under the conditions of a span of 12 mm and a crosshead speed of 1.0 mm/s. The specimen was positioned so that the glaze layer was the tensile surface.

The three-point flexural strength was calculated according to the following equation [22]:

(1) $$\sigma =\frac{3\mathrm{Pl}}{2wt^{2}},$$

*P*: breaking load [N];

*l*: test span [mm];

*w*: width of the specimen [mm];

*t*: thickness of the specimen [mm].

After the test, the appearance and the fractured surface of each test piece were observed with an optical microscope (VHX-5000, Keyence, Osaka, Japan), and the thickness of the glaze layer was measured.

### 2.3. Finite Element Method (FEM) Analysis

**Figure 2:**
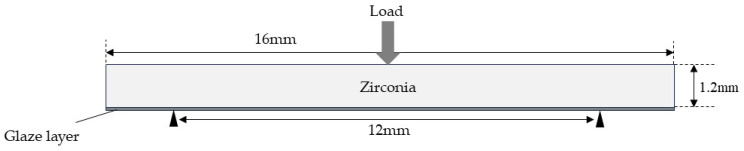
FEM analysis model.

FEM analysis was performed using open-source FEM software Code_Aster (version number: 14.6) to calculate the stress distribution during three-point flexural tests on glazed specimens. A model in which a glaze layer (thickness: 0, 10, 40, and 100 μm) was placed on a base material (16.0 mm × 4.0 mm × 1.2 mm) was used for the analysis (Figure 2). The Young’s modulus of each material used in the analysis is 210 GPa [23] (zirconia) and 61 GPa (glaze). All models were considered to be isotropic, homogeneous, linear elastic, and meshed with tetrahedral elements. Boundary conditions were set at the contact points between the specimen and the support rods to prevent displacement in the z-direction (load direction). The load was set as a line load perpendicular to the specimen’s long axis.

### 2.4. Fracture Surface Observation

The fracture surface of the sample was coated with Au to prevent charge-up and observed using a scanning electron microscope (SEM; JSM6390LA, JEOL, Tokyo, Japan) at an acceleration voltage of 15 kV. The fracture surface features were analyzed in accordance with previously reported guidance [24], and the fracture origin, crack propagation direction, and hackle line were estimated.

### 2.5. Raman Spectroscopy

Raman spectra of glazed zirconia specimens were measured to investigate the monoclinic content of the zirconia-glaze layer interface. Measurements were performed using a laser Raman spectrometer (T64000, HORIBA, Toyo, Japan) using a laser with a wavelength of 532 nm as the excitation source, focusing on the zirconia surface on the glazed side of a glazed 3Y zirconia flexural specimen without grinding or polishing.

In order to investigate the amount of tetragonal → monoclinic phase transformation on the fracture surface of the specimen, the Raman spectrum of the fracture surface of the glazed 3Y zirconia specimen was measured using laser Raman microscope (RAMANtouch, Nanophoton, Osaka, Japan) using a laser with a wavelength of 532 nm as the excitation source. Measurements were carried out in 4.14 μm increments in a 1693 μm × 768 μm area, including the fracture origin, and a map of the monoclinic peak intensity around 176 cm^−1^ was created. The monoclinic content was calculated from the average spectrum in the range of 765.36 µm height × 1654.83 µm width from the fracture origin using Equation (2) [25].

(2) $$V_{m}=\frac{I_{m}^{176}+I_{m}^{183}}{0.97\left(I_{t}^{144}+I_{t}^{256}\right)+I_{m}^{176}+I_{m}^{183}}$$

here, *I* indicates the peak intensity, and the subscripts m and t indicate the monoclinic phase and tetragonal phase, respectively.

### 2.6. Statistical Analysis

A two-way analysis of variance (two-way ANOVA) and Tukey–Kramer multiple comparison tests were used to compare the three-point flexural strength of the control and removal groups of various zirconia. A Tukey-Kramer multiple comparison test was used to investigate the effect of glaze thickness on the three-point flexural strength of various glazed zirconia specimens. The strengths of specimens with three glaze thickness ranges were compared.

## 3. Results

### 3.1. Appearance Observation of the Fractured Specimen

**Figure 3:**
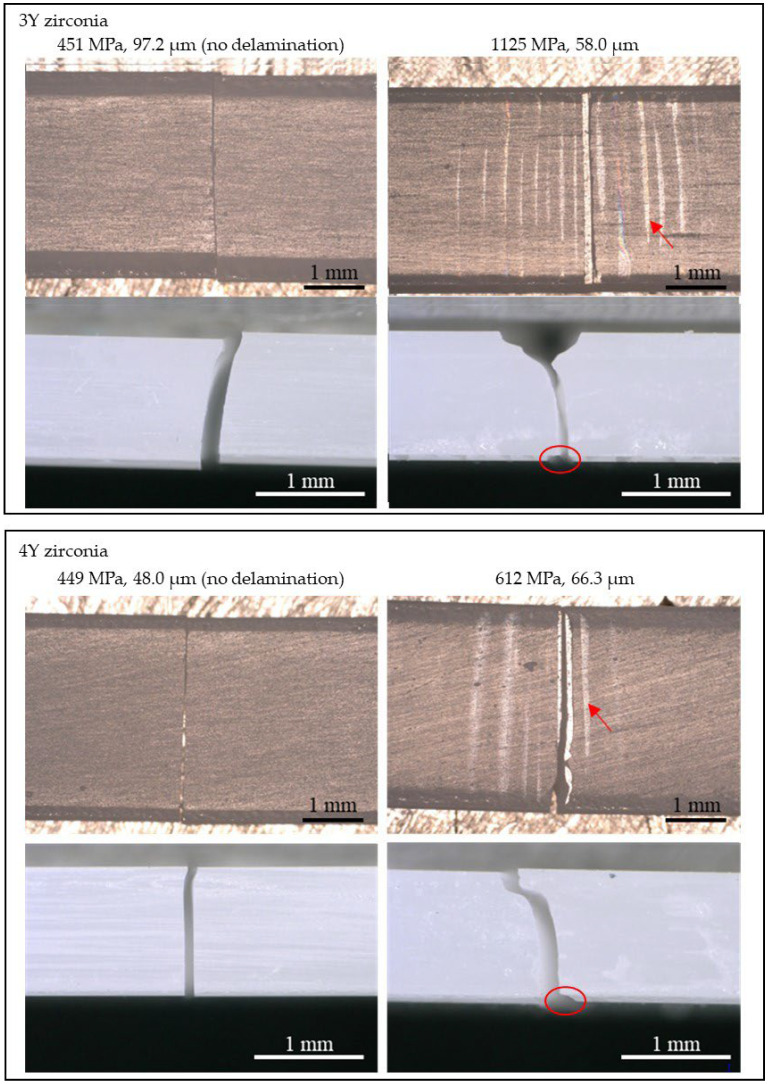
Photomicrographs of fractured 3Y and 4Y zirconia specimens. The top and bottom are porcelain surface and side views, respectively. The arrows indicate channel cracks. The red circles indicate delamination of the glaze material.

For 5Y zirconia, no delamination of the glaze layer was observed in any of the test specimens. On the other hand, for 3Y zirconia and 4Y zirconia, delamination of the glaze layer was observed in test specimens with glaze thicknesses of >44 μm and >54 μm, respectively. In particular, for 3Y zirconia with a glaze layer of 100 μm or more, the glaze layer delaminated in all six test specimens. Optical microscope photographs of representative test specimens with a delaminated glaze layer are shown in Figure 3. The photograph from the surface of the glaze layer shows that multiple channel cracks have occurred and that delamination occurred from the channel cracks (bright areas). The photograph from the side of the test specimen shows that the cracks deflected at the glaze layer–zirconia interface, and as the delamination progressed, the cracks kinked and entered the zirconia.

### 3.2. FEM Analysis

**Figure 4:**
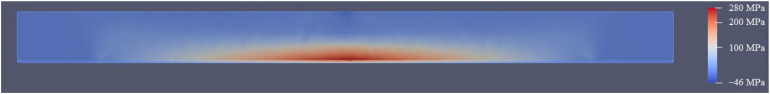
Principal stress distribution calculation results in three-point flexural test using FEM (glaze thickness: 40 μm). The red area indicates stress concentrations.

**Figure 5:**
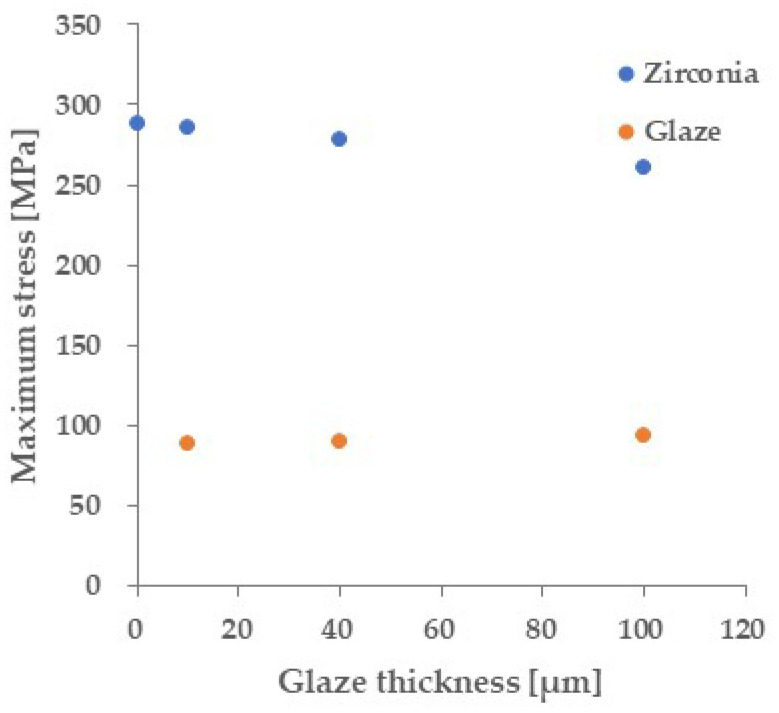
Calculated maximum stress applied to the base material and glaze layer during a three-point flexural test (Load: 100N). The stress distribution in each material changes depending on the glaze thickness.

**Figure 6:**
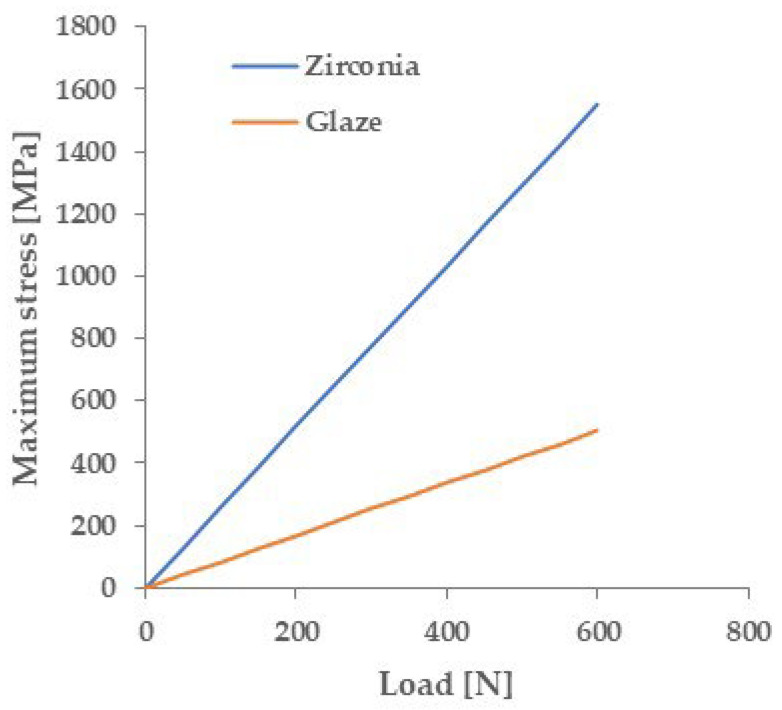
Calculated maximum stress applied to the base material and glaze layer during a three-point flexural test (glaze thickness: 40 μm).

Figure 4 shows the primitive stress distribution during a three-point flexural test on a glazed specimen using FEM. The maximum tensile stress was concentrated not on the glaze surface, which is the tensile surface, but on the zirconia surface inside the specimen. Figure 5 shows the relationship between the maximum tensile stress applied to the glaze surface and the zirconia surface, respectively, and the glaze layer thickness. The tensile stress on the zirconia surface was greater than that on the glaze surface, regardless of the glaze layer thickness. Figure 6 shows the relationship between the stress applied to each material and the flexural load when the glaze thickness is 40 μm, calculated by FEM. A flexural load of 144 N causes a stress of 129 MPa (≈ flexural strength of the glaze material) in the glaze layer. On the other hand, under this load, zirconia is applied to a stress of 372 MPa. This stress is significantly lower than the flexural strength of any type of zirconia.

### 3.3. Three-Point Flexural Test

**Figure 7:**
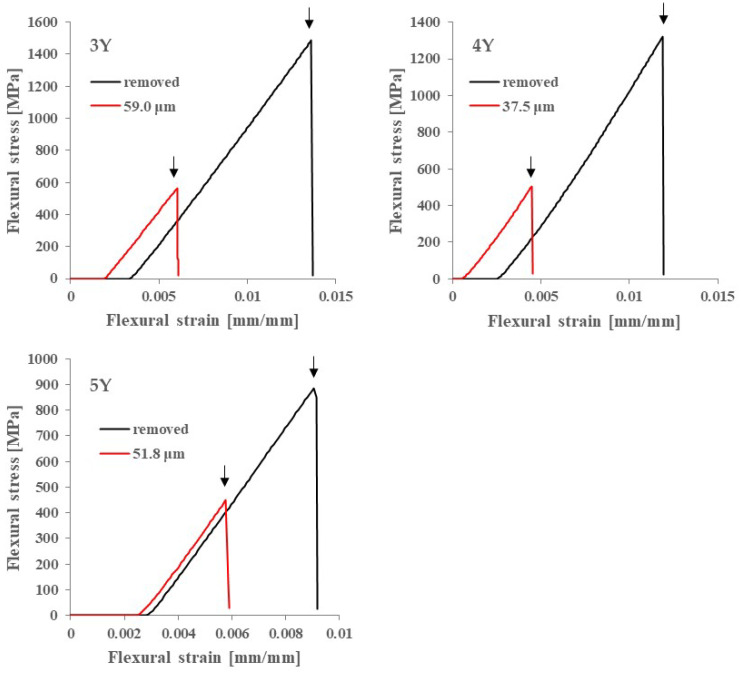
Representative stress–strain curves for each specimen. The arrows in Figure 7 indicate the fracture points.

Figure 7 shows typical stress–strain curves for each specimen. All specimens exhibited typical brittle material characteristics, and the shape and slope of the curves did not change with or without the glaze layer. Table 1 shows the three-point flexural strength of the glaze-removed group and the control group. Table 2 shows the results of the two-way ANOVA statistical analysis. A significant difference was observed only in the type of base material, and no significant difference was observed for the presence or absence of the glazing → removal procedure.

**Figure 8:**
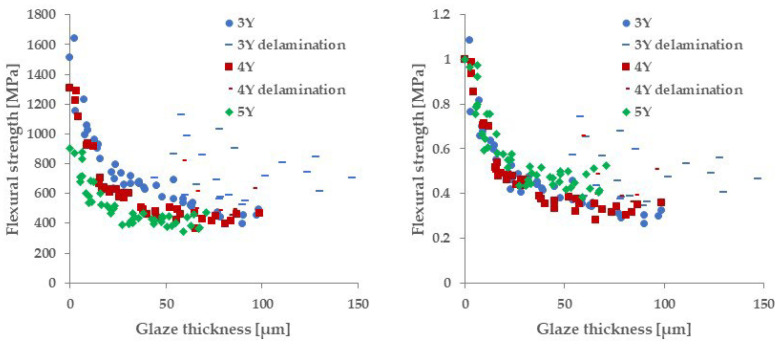
Relationship between flexural strength calculated from Equation (1) and glaze thickness of each material. The figure on the right shows the measured values normalized by the glaze-removed strength of each material. The bar-shaped elements are specimens in which the glaze material delaminated during the test and deviated from the strength reduction curve.

The left side of Figure 8 shows the relationship between the three-point flexural strength and glaze thickness for each specimen. In any of the base materials, the three-point flexural strength tended to decrease as the thickness of the glaze layer increased. The right side of Figure 8 shows the dependence of the strength on the glaze layer thickness, normalized by the strength of the glaze-removed group. All base materials showed similar strength reduction curves. On the other hand, the delaminated specimens deviated above these curves, and no strength dependency on the glaze thickness was observed.

**Figure 9:**
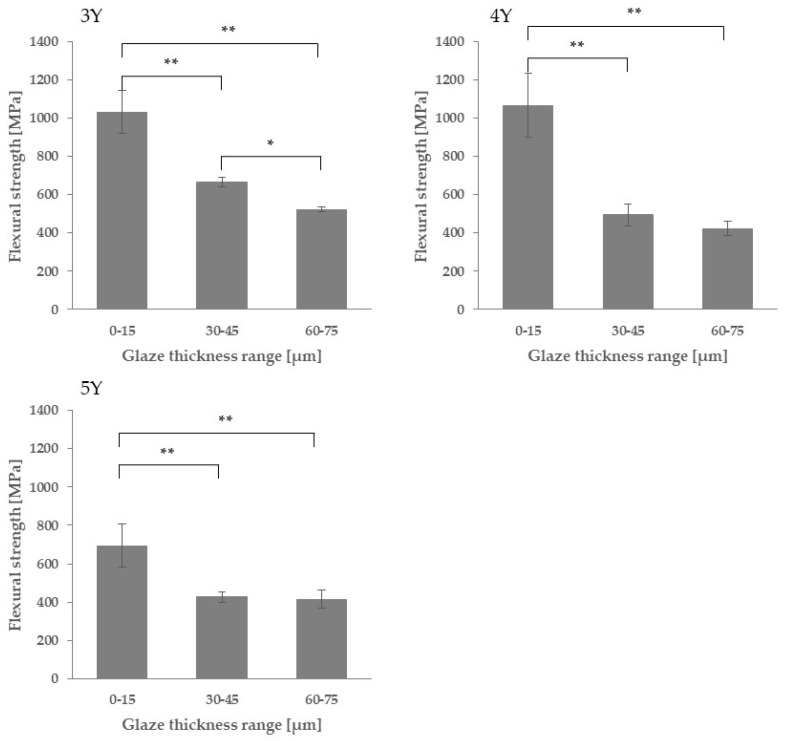
Average three-point flexural strength for each glaze thickness range. The asterisks refer to statistical significance according to the Tukey–Kramer multiple comparison test (*: *p* < 0.05, **: *p* < 0.01).

Figure 9 shows the three-point bending strength of each zirconia material in the three glaze thickness ranges. For 3Y zirconia, statistically significant differences were observed between all glaze thickness ranges. For 4Y and 5Y, statistically significant differences were observed between the 0–15 μm group and the 30–45 μm and 60–75 μm groups, but no significant difference was observed between the 30–45 μm group and the 60–75 μm group.

**Figure 10:**
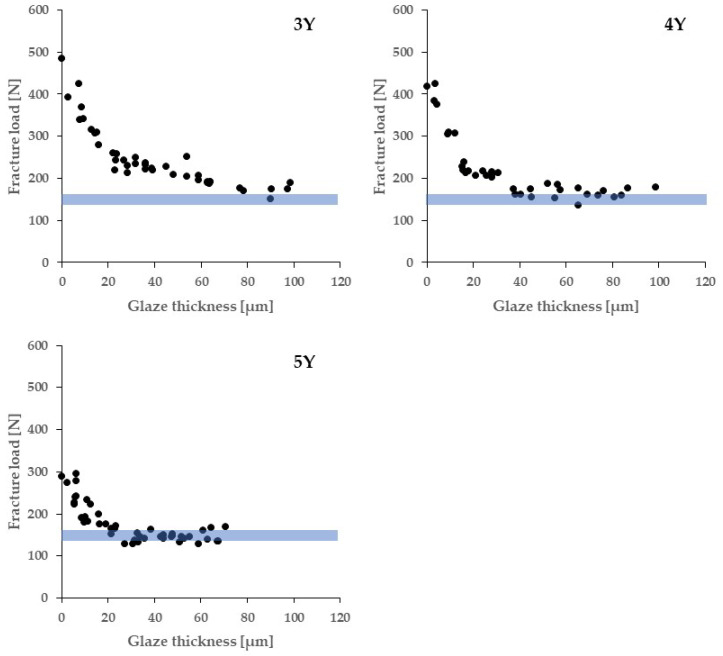
Relationship between fracture load and glaze thickness of each material (data from delaminated specimens were excluded). The blue shading indicates the load range at where the fracture stress of the glaze material is applied to the glaze layer, estimated from Figure 6.

The three-point flexural strength in Figure 8 is calculated by Equation (1), which shows that in a three-point flexural test, the maximum stress on the tensile surface is inversely proportional to the square of the specimen thickness. Therefore, the maximum stress decreases by about 15% when the specimen thickness increases from 1.2 mm to 1.3 mm. On the other hand, Figure 5 shows that the maximum stress on the tensile surface (glazed surface) of the glazed specimen instead increases by about 6%. In this test range (glaze thickness: 0 to 100 μm). That is, the strength calculated from Equation (1) is underestimated compared to the actual strength, especially for specimens with large glaze thickness. Therefore, in order to discuss the influence of the glaze layer more accurately on strength, Figure 10 shows the relationship between fracture load and glaze thickness. The blue shading indicates the load range at where the fracture stress of the glaze material is applied to the glaze layer, estimated from Figure 6. The upper (158 N) and lower (132 N) limits of this load range were calculated using the maximum and minimum fracture stresses in the three-point flexural test of the monolithic glaze material. This Figure 7 gain shows the dependence of strength on glaze thickness. Additionally, this figure shows that the decrease in the fracture load with increasing glaze thickness stops at a load where the glaze material reaches a fracture stress.

### 3.4. Fracture Surface Observation

**Figure 11:**
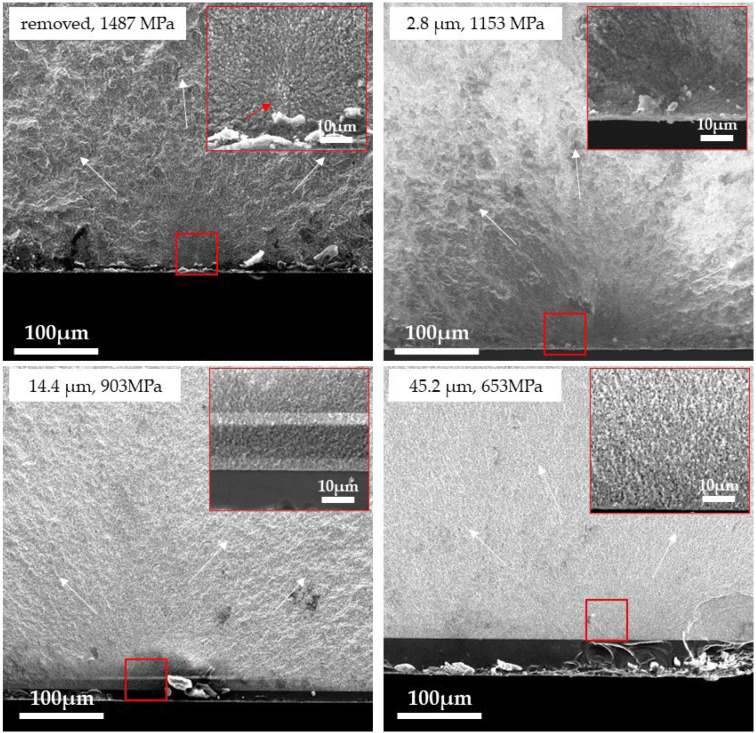
SEM image of the fracture surface of the 3Y zirconia specimen. The white arrows indicate the direction of crack propagation. The inset shows a high-magnification image of the area outlined in red. The red arrow indicates the fracture origin.

**Figure 12:**
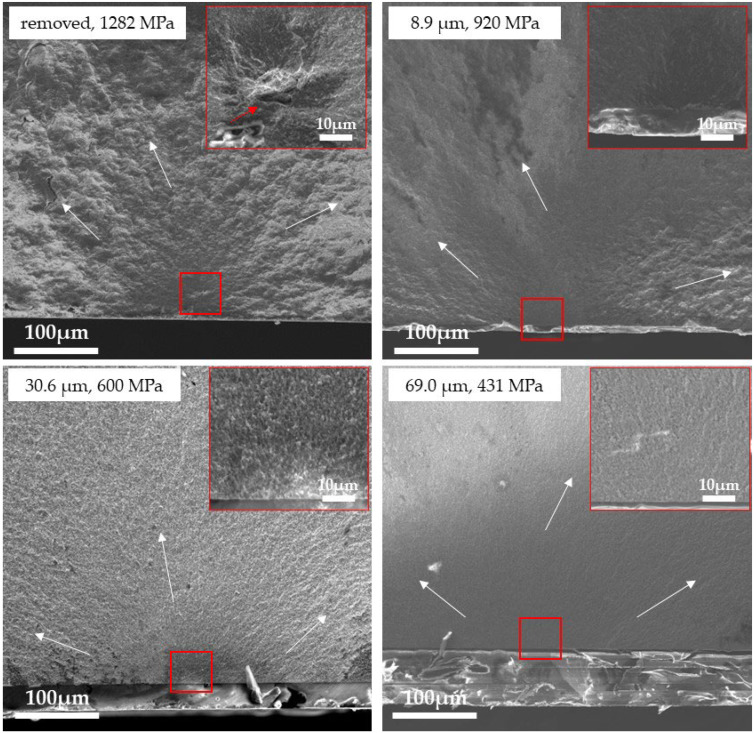
SEM image of the fracture surface of the 4Y zirconia specimen. The white arrows indicate the direction of crack propagation. The inset shows a high-magnification image of the area outlined in red. The red arrow indicates the fracture origin.

**Figure 13:**
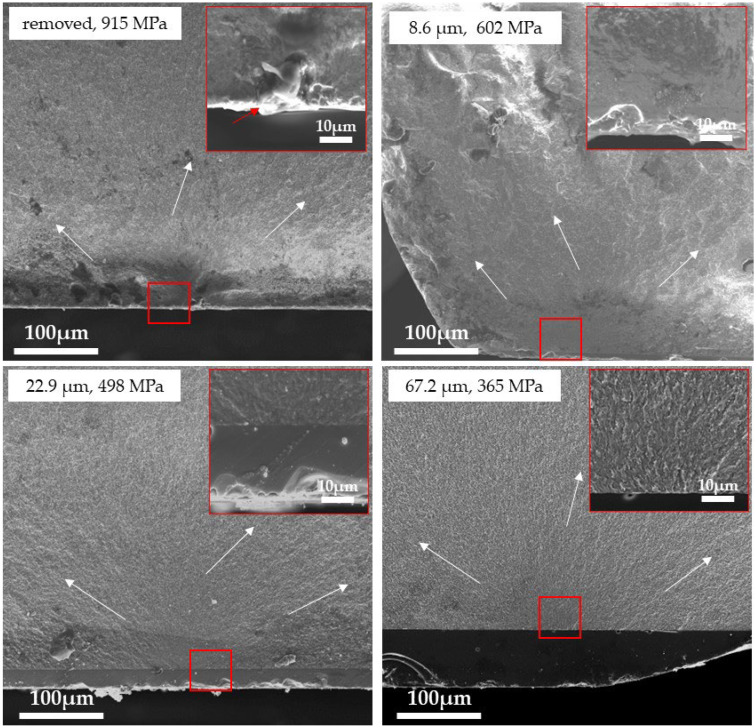
SEM image of the fracture surface of the 5Y zirconia specimen. The white arrows indicate the direction of crack propagation. The inset shows a high-magnification image of the area outlined in red. The red arrow indicates the fracture origin.

**Figure 14:**
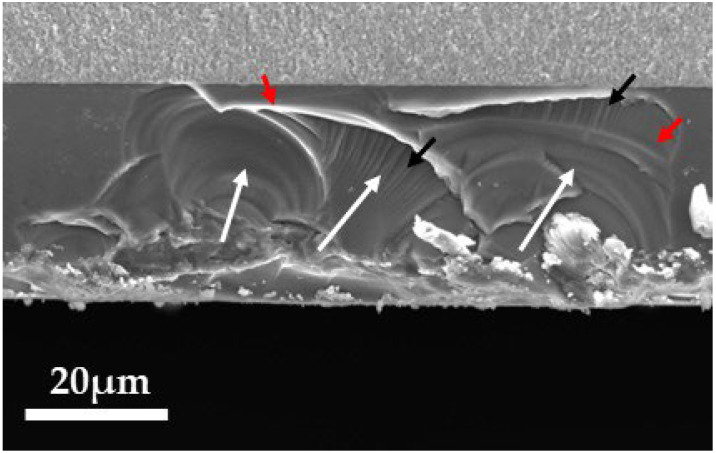
SEM images of the fracture surface of the glaze layer on a 3Y zirconia specimen (glaze thickness: 45.2 μm, three-point flexural strength: 653 MPa). The white, black, and red arrows indicate the direction of crack propagation, hackles, and rib marks, respectively.

Figure 11, Figure 12 and Figure 13 show the fracture surfaces of the 3Y, 4Y, and 5Y zirconia specimens (without delamination) observed by SEM, respectively. In all specimens, the glaze layer and the base layer were completely adhered to, and no delamination was observed. Hackles were observed in all samples, and the hackle undulations tended to decrease as the fracture stress decreased (as the glaze thickness increased). In the removed specimens, defects that were the fracture origins (3Y: insufficiently densified area, 4Y: pore, and 5Y: coarse grain) were clearly observed in the center of the mirror area, but in all glazed specimens, typical fracture origins could not be identified in the base materials. The fracture surface of the glaze layer is shown in Figure 14. Hackle lines and rib marks were observed on the fracture surface of the glaze layer, and the direction of crack propagation was estimated.

### 3.5. Raman Spectroscopy

**Figure 15:**
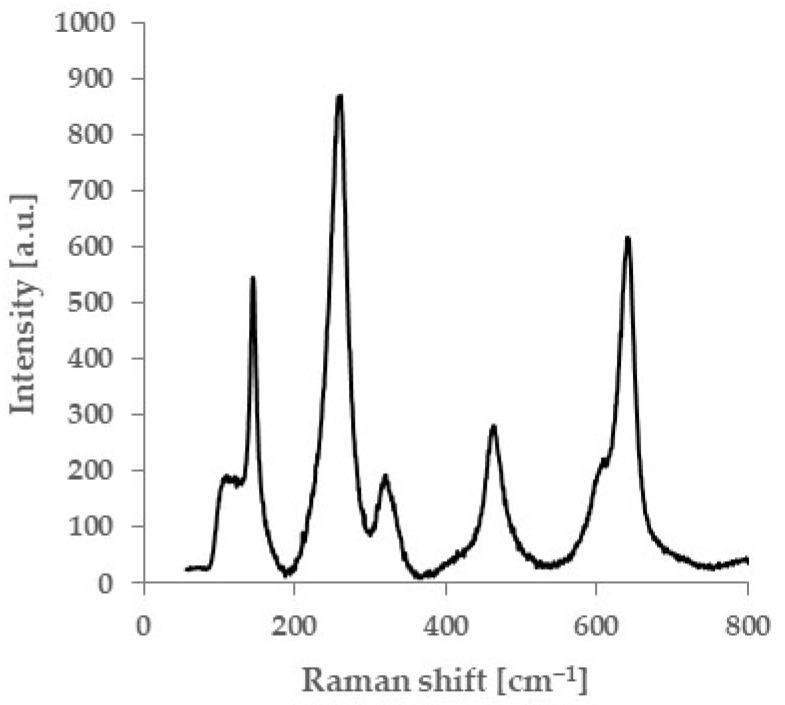
Raman spectrum on glazed 3Y zirconia surface. No typical peaks indicative of the monoclinic phase (176 cm^−1^ and 183 cm^−1^) are observed.

**Figure 16:**
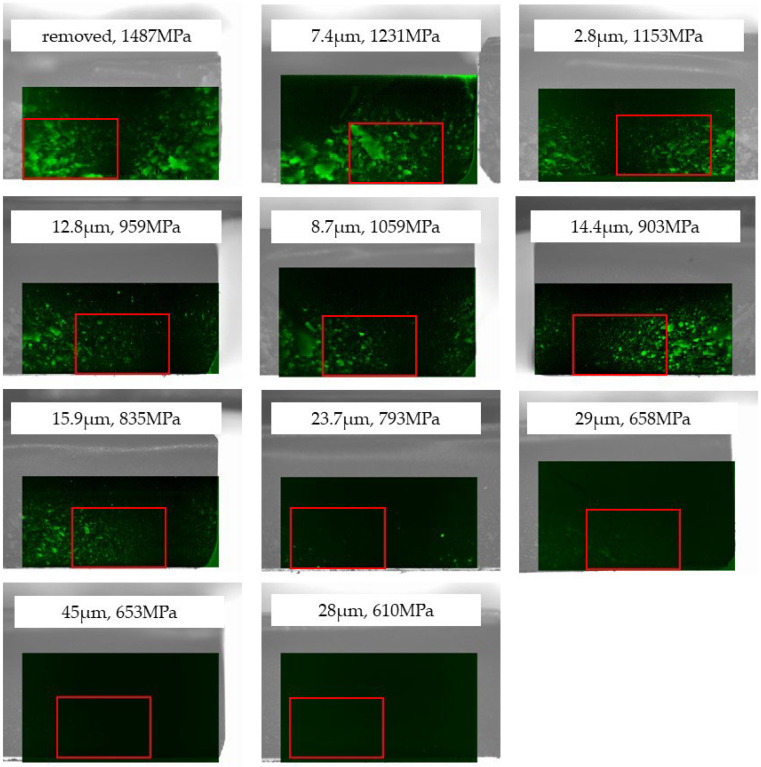
Map of the monoclinic peak (~176 cm^−1^) intensity on the fracture surface of glazed 3Y zirconia. The red line indicates the area where the average monoclinic phase content was calculated (Figure 17).

Figure 15 shows a Raman spectrum focused on the glaze material–zirconia interface of a glazed 3Y zirconia specimen. The glazed test specimen showed a Raman peak attributed to tetragonal zirconia, while no Raman peak derived from the monoclinic phase was observed. Figure 16 shows a map of monoclinic peak intensity obtained by Raman spectroscopy of the fracture surface of glazed 3Y zirconia. A fraction of the monoclinic phase was present on the fracture surfaces of specimens with flexural strengths between 1500 MPa and 835 MPa, whereas no monoclinic phase was observed in specimens with lower flexural strengths.

**Figure 17:**
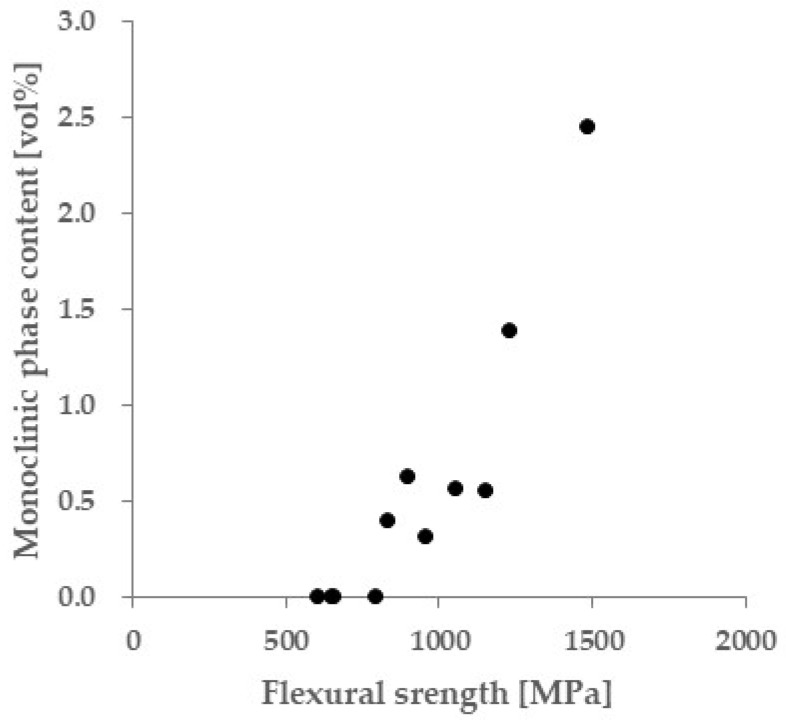
Relationship between average monoclinic phase content of fracture surface calculated from Equation (2) and flexural strength of glazed 3Y zirconia.

Figure 17 shows the average monoclinic fraction for the region, as shown in Figure 16. The monoclinic ratio decreased as the specimen strength decreased and was zero below 835 MPa.

## 4. Discussion

In this study, three-point flexural tests were carried out on zirconia specimens with glaze layers of various thicknesses to clarify the effect of glaze thickness on the strength of various types of zirconia. Figure 9 shows that the strength significantly decreases with increasing glaze thickness for all zirconia types. Therefore, the null hypothesis is rejected. In the following subsections, the mechanism is discussed in terms of the chemical and mechanical effects of glazing, crack propagation, and stress-induced phase transformation of zirconia.

### 4.1. Chemical and Crystallographical Effect of Glazing

The stress–strain curves of all specimens exhibited typical characteristics of brittle materials, and no change in slope was observed depending on the presence or absence of the glaze layer. This result is consistent with previous report [13]. The three-point flexural test results showed that glazing reduced strength for all base materials (Figure 8). One possible cause of strength reduction due to glazing could be changes in the chemical composition and crystal structure of the base material. Lunt et al. reported that in porcelain fused zirconia, the tetragonal phase transformed into the monoclinic phase in a region 10 μm from the zirconia surface [26]. This phase transformation is caused by the tensile stress applied to the zirconia surface due to the difference in thermal expansion between the zirconia and the porcelain. Such phase transformation can reduce strength by causing microcracks and particle detachment [27]. On the other hand, in this study, no monoclinic phase was detected on the zirconia surface (Figure 15). This discrepancy may be due to differences in glaze thickness. In a study by Mainjot et al. [28], when a 1.5 mm thick porcelain layer was formed on a 1.0 mm thick zirconia plate, tensile stress suggesting a T→M phase transition of zirconia was detected near the interface of the porcelain layer. Meanwhile, it was shown that compressive stress was detected when the porcelain layer thickness was 1.0 mm. The reason for this change in residual stress was explained to be that the reduced thickness of the porcelain layer reduces the tensile stress on the zirconia due to shrinkage of the porcelain during its solidification process. In this study, the thickness of the porcelain layer before polishing was approximately 0.2 mm, so the tensile stress on the zirconia during glazing was smaller than in the case of Lunt et al. [26] and Mainjot et al. [28] Therefore, the T→M phase transition would not have occurred.

In addition, Lunt et al. showed that in porcelain fused zirconia, constituent elements (Na, Al, Si, and K) of the porcelain were detected in a region several micrometers from the zirconia surface [26]. The doping effect of alkali metal ions on zirconia has not been reported to our knowledge. On the other hand, the addition of trace amounts of silica and alumina is known to improve strength [29,30]. If the diffusion of these elements affects the strength of zirconia, then the strength of the glaze-removed group should also change. However, in this study, the strength of the glaze-removed group was comparable to that of the control group. Therefore, even if diffusion of these elements into zirconia occurs, it is considered that it does not affect the strength.

As described above, the decrease in strength due to glazing is not a chemical phenomenon and is therefore considered to be due to mechanical effects.

### 4.2. Mechanical Effect of Glazing

In Section 4.1, it was revealed that the chemical interaction between the glaze material and zirconia does not affect the strength. Here, the mechanical effects of glazing are discussed. The strength decreases of zirconia due to glazing has been reported by several researchers [10,11,12,13,14,15,16]. Possible causes are the surface roughness of zirconia and residual stress due to the difference in the CTE between zirconia and glaze material. In the study by Doğru et al. [12], the zirconia surface in the glazed group was not polished, so the difference in surface roughness compared to the control group (with surface polishing) may have led to the difference in strength. However, in this study, the same zirconia surface polishing protocol was used in the control and glazed groups, so the strength decrease was not due to the zirconia surface roughness. On the other hand, the residual stress calculation by Swain et al. [19] showed that the residual stress of the zirconia surface is about 40 MPa, even in zirconia with a 3 mm thick porcelain layer. Therefore, the significant strength decrease in this study cannot be explained by the residual stress due to the difference in the CTE. These facts suggest that the presence of the glaze layer itself reduces the strength of zirconia.

### 4.3. Crack Initiation and Propagation

In order to clarify where fracture begins, the stress applied to the glaze layer and base material during the three-point flexural test was calculated using FEM analysis. FEM analysis showed that the tensile stress applied to the base material is 2.8–3.3 times larger than to the glaze layer. (Figure 5). Based on the report by Hsue et al. [31], Lobo et al. reported that the stress applied to the zirconia base material in a biaxial flexural test was approximately four times larger than to the glaze layer [32]. Huang et al. showed by FEM analysis that the maximum stress was applied to the zirconia surface in a biaxial flexural test of a specimen with a 0.9 mm porcelain layer placed on 0.9 mm zirconia [33]. The calculation results of this study are consistent with these reports. The reason greater stress was applied to the inner base material than to the bottom glaze layer is mainly due to the difference in the stiffness of the materials. In bi-materials, stress is concentrated in the stiffer material, so if a stiffer substrate (or less stiff glaze material) is used, the stress concentration in the substrate will increase.

The relationship between the stress applied to each material and the flexural load calculated by FEM (Figure 6) showed that when the fracture stress of the glaze material was applied to the glaze layer, the stress on the zirconia was significantly lower than the fracture stress of any type of zirconia material. This result suggests that fracture occurs from the glaze layer in the three-point flexural test.

Observation of the fracture surface (Figure 11, Figure 12 and Figure 13) showed that the fracture origins of the deglazed samples were insufficiently densified area (3Y zirconia), pore (4Y zirconia), and coarse grain (5Y zirconia). These are typical fracture origins for zirconia ceramics [24]. On the other hand, typical fracture origins were not observed in the glazed sample. This result indicates that defects near the zirconia surface are not the origin of the fracture in the glazed sample. Hackles and rib marks were observed in the glaze layer (Figure 14), suggesting that cracks were propagating from the surface of the glaze layer toward the zirconia layer. These results indicate that the fracture of the glazed sample started from the glaze layer, and the crack propagated to the zirconia layer, which is consistent with the stress distribution calculated by FEM.

### 4.4. Dependence of Strength on Glaze Thickness

#### 4.4.1. Mechanism of Strength Decrease with Increasing Glaze Layer Thickness

As discussed in Section 4.2, the failure of the glazed group is considered to have started from the surface of the glaze layer. The cracks that were generated reached the interface between the glaze material and zirconia almost simultaneously [34]. Mukherjee et al. [35] theoretically demonstrated that the stress intensity factor of the crack tip propagating from the tensile surface of a bilayer material in a four-point bending test is proportional to the square root of the crack length. However, this relationship does not consider the state where the crack reaches the interface. It is difficult to quantitatively understand the stress intensity factor at the crack tip when the crack reaches the interface. Since the stress distribution of the bilayer material in the bending test is significantly different between the zirconia side and the glaze side of the interface (the stress on the glaze layer decreases as it approaches the interface, while the stress on the zirconia is maximum at the interface [18]). However, unless the glaze material shows R-curve behavior (such as bridging or stress-induced phase transition), the qualitative relationship between crack length and the stress intensity factor should be maintained. That is, the longer the crack, the larger the stress intensity factor at the crack tip. From these discussions, the dependence of the fracture load on the glaze thickness is thought to be due to stress concentration at the crack tip that has propagated from the glaze layer to the base material. That is, the thicker the glaze layer is, the longer the crack length is when it reaches the substrate. Since the stress concentration at the crack tip increases as the crack becomes longer, the fracture load decreases as the glaze layer becomes thicker.

#### 4.4.2. Region with Constant Fracture Load

The fracture loads of 4Y and 5Y zirconia became constant in the thick glaze region at a specific load (shaded region in Figure 10). At this load, the stress in the glaze layer reaches the fracture stress of the monolithic glaze material. Therefore, the glaze layer is not fractured below this load. As a result, the fracture load becomes a constant value after specific glaze thicknesses (4Y: 40 μm, 5Y: 30 μm). Similarly, for 3Y zirconia, the strength decreased as the glaze thickness increased, and the slope of the curve became smaller as it approached the shaded region. If the glaze thickness was increased further, it was predicted that the fracture load would become constant near the shaded region, as with 4Y and 5Y; however, this could not be verified because delamination occurred in all specimens with a glaze thickness of >100 μm.

To summarize the above discussion, in a three-point flexural test of a glazed specimen, cracks propagate from the glaze layer and reach the interface with the base material. The stress at the crack tip increases as the crack length (i.e., glaze thickness) increases, so the fracture load decreases. On the other hand, if the load is less than 132 N, no cracks will occur in the glaze layer, so the material will not be fractured. Therefore, above a certain glaze thickness, the fracture load plateaus at the point.

### 4.5. Tetragonal-to-Monoclinic Phase Transformation

From the measured Raman spectra, the tetragonal- to-monoclinic phase transformation of zirconia is discussed. Figure 13 and Figure 16 show that a monoclinic phase exists on the fracture surface of specimens only with flexural strength of 835 MPa or higher. The phase transformation occurred along the hackle, indicating that large local stress was applied during hackle formation. A hackle is a fracture surface feature that occurs when cracks branch out when fracture occurs rapidly due to large stresses [24,36]. The phase transformation from tetragonal to monoclinic is accompanied by a volumetric expansion of approximately 4%. Therefore, when a phase transformation occurs as a crack develops, stress is generated to close the crack, suppressing the growth of the crack (transformation toughening) [37,38,39,40,41]. The detection of a monoclinic phase on the fracture surface suggests that transformation toughening occurred. However, since the amount of phase transformation is small, the effect of phase transformation on the strength of the test specimen is expected to be limited. In fact, 4Y and 5Y zirconia, which are less prone to phase transformations, show almost the same strength reduction curves as 3Y zirconia (Figure 3).

### 4.6. Delamination of Glaze Layer

In this study, multiple cracks in the glaze layer and delamination at the interface were observed in some of the 3Y and 4Y zirconia specimens. Many researchers have investigated the delamination of bilayer materials theoretically and experimentally. Chai et al. [34] investigated the initiation and propagation of cracks in four-point bending tests of zirconia materials with porcelain layers. In their report, a crack was first initiated on the porcelain layer surface (tensile surface) and stopped at the zirconia interface, and a new channel crack was generated at a slightly higher load, and then the delamination progressed. The multiple channel cracks and delamination observed in this study are consistent with the report by Chai et al. In this study, delamination of the glaze layer was observed only in 3Y and 4Y zirconia. In bilayer materials, whether a crack that reaches the material interface penetrates or deflects is determined by the energy release rate of penetration, the energy release rate of deflection, and the residual stress [42]. The fracture toughness varies greatly depending on the type of zirconia, and 3Y zirconia shows the highest fracture toughness. Therefore, the energy release rate of penetration of 3Y and 4Y zirconia is larger than that of 5Y zirconia. On the other hand, the contribution of the energy release rate of deflection and the residual stress can be estimated from the adhesive strength between the substrate and porcelain. Dimitriadis et al. [43] reported that the yttria concentration of zirconia does not affect the bond strength between zirconia and porcelain, and it is considered that the energy release rate of deflection hardly changes depending on the type of zirconia. Therefore, zirconia with a low yttria concentration has a relatively low energy release rate at the interface, and delamination is more likely to progress. These are the reasons why delamination occurred only in 3Y and 4Y zirconia. Delamination of the glaze layer occurred only in specimens with a large glaze layer thickness (3Y zirconia: >44 μm, 4Y zirconia: >54 μm). Wang et al. [44] showed that in a bilayer material of zirconia and porcelain, the energy release rate of the interface crack decreases as the thickness of the porcelain layer increases. This is because the thicker the porcelain layer, the smaller the ratio between the shear and tensile modes at the interfacial crack tip. In other words, in the specimens with a small porcelain layer, the opening stress for the interfacial crack was small, so delamination did not progress.

### 4.7. Clinical Impact

The fact that glazes significantly reduce the strength of dental ceramic materials may be of clinical concern. In clinical practice, the thickness of the glaze layer varies greatly depending on the operator, but it is estimated to be approximately several tens of micrometers to 100 μm (a thickness of 50 μm or more is reported to be sufficient to preserve the integrity of the glaze layer [45]). In this thickness range, the strength of zirconia is 3Y: 500 MPa to 800 MPa, 4Y: 450 MPa to 600 MPa, and 5Y: 400 MPa to 500 MPa (Figure 2). ISO 6872 [22] specifies the minimum strength required in each case. The required strength for crowns or 3-unit bridges on anterior teeth is 300 MPa or more, so even when using 5Y zirconia, it can be used in these cases. For a 3-unit bridge that includes the molars, the material must have strength of 500 MPa or more. In many cases, 3Y or 4Y zirconia is used for molar bridges, so even if glazing is used, the standards will be met. One may need to be careful when creating a bridge with more than 4 units. Materials used in bridges with four or more units require a strength of 800 MPa or more. In order to maintain such a strength level in 3Y zirconia, the glaze thickness must be 20 μm or less. However, it must be noted that the above discussion is based only on test results with simple geometries. Since various factors overlap in actual clinical practice, it is difficult to discuss the reliability of actual prosthetic devices based only on the results of this study. Further clinically useful information will be obtained by combining the results of this study with stress analysis using FEM. Previous studies showed that large stresses are concentrated on prosthetic devices using FEM (e.g., the connector part of a bridge) [46,47]. In clinical practice, applying a thick glaze layer to such areas is avoided, but there are no quantitative guidelines. The present study provided basic information on the relationship between glaze layer thickness and zirconia strength. By combining this with stress analysis using FEM, a guideline for which areas and how thick the glaze layer should be in an actual prosthesis is provided. This should lead to increased reliability of dental prostheses. In addition, a more practical understanding will be obtained through calculations and experimental evaluations using shapes and loading conditions that correspond to actual clinical practice, as well as evaluations of fracture toughness and fatigue properties.

## 5. Conclusions

Three-point bending strength tests of glazed zirconia revealed that the strength (fracture load) of all types of zirconia decreased with increasing glaze thickness. To clarify the mechanism behind this decrease in strength, FEM analysis and fracture surface observations were performed, which showed that stress concentration at the tip of a crack propagating from the glaze material caused the strength decrease in zirconia. At loads below 150 N, the glaze material did not break, so the decrease in fracture load stopped at this value. Since glazes of various thicknesses are applied in clinical practice, it is expected that the relationship between glaze layer thickness and zirconia strength revealed in this study will contribute to improving the reliability of prosthetics. The limitation of this study is that only bending tests were performed on simple geometries. Real prostheses have much more complex geometries, and more complex stress distributions are expected. In addition, fracture toughness and fatigue properties, which are important for evaluating the reliability of prostheses, were not evaluated. These further investigations would be very useful in clinical practice.

## Figures and Tables

**Table 1:** Three-point flexural strengths of each material.

		3Y	4Y	5Y	GP
Control	Flexural strength	1502.2 ^A,a^	1301.9 ^B,b^	919.7 ^C,c^	129.2 ^D^
	Standard deviation	169.0	125.8	121.1	6.9
Glaze-removed	Flexural strength	1511.7 ^A,a^	1294.2 ^B,b^	901.2 ^C,c^	—
	Standard deviation	120.2	126.5	65.4	—

Similar letters indicate a lack of statistical difference (*p* > 0.05). Uppercase letters indicate statistical differences between materials. Lowercase letters indicate statistical differences with and without the glazing → removal procedure.

**Table 2:** Results of two-way ANOVA statistical analysis of three-point flexural test.

Source	SS	MS	F	*p*	*: *p* < 0.05 **: *p* < 0.01
Material	3,664,159.80	1,832,079.90	117.1856	<0.001	**
Glazing → removal procedure	459.56	459.56	0.0294	0.8645	
Material * Glazing → removal procedure	1989.51	994.76	0.0636	0.9384	
Error	844,236.02	15,634.00			
Total	4,510,844.89				

SS: Sum of squares; MS: Mean square; F: F value; *p*: *p* value.

## Data Availability

The original contributions presented in this study are included in the article. Further inquiries can be directed to corresponding author.
